# Direct Oral Anticoagulant Monotherapy Versus Dual Antiplatelet Therapy After Left Atrial Appendage Closure: A Meta-Analysis of Randomized Trials

**DOI:** 10.1161/CIRCINTERVENTIONS.126.016554

**Published:** 2026-05-20

**Authors:** Nicola Ammirabile, Daniele Giacoppo, Placido Maria Mazzone, Davide Landolina, Davide Capodanno

**Affiliations:** 1 Division of Cardiology, Azienda Ospedaliero-Universitaria Policlinico G. Rodolico-San Marco, University of Catania, Italy (N.A., D.G., P.M.M., D.C.).; 2 Cardiology Unit, Umberto I Hospital, Siracusa, Italy (D.L.).

**Keywords:** anticoagulants, atrial fibrillation, hemorrhage, left atrial appendage closure, meta-analysis, platelet aggregation inhibitors, stroke

The optimal short-term antithrombotic regimen after left atrial appendage closure (LAAC) remains uncertain, reflecting the need to balance thromboembolic prevention against bleeding risk. Although dual antiplatelet therapy (DAPT) is commonly used for 1 to 3 months after LAAC, observational data indicate that direct oral anticoagulant (DOAC) monotherapy may provide a more favorable balance between thromboembolic and bleeding events after LAAC.^1^ Against this background, we performed a systematic review and meta-analysis of randomized clinical trials comparing DOAC monotherapy with short-term DAPT following transcatheter LAAC to define major clinical outcomes and net clinical benefit between these antithrombotic strategies (PROSPERO [International Prospective Register of Systematic Reviews]; URL: https://www.crd.york.ac.uk/PROSPERO/view/CRD420251242122; Unique identifier: CRD420251242122).

We identified 3 randomized trials comparing DOAC monotherapy with DAPT after LAAC at 60 to 90 days: ADRIFT trial (Assessment of Dual Antiplatelet Therapy Versus Rivaroxaban in Atrial Fibrillation Patients Treated With Left Atrial Appendage Closure), ADALA trial (Apixaban Versus Dual Antiplatelet Therapy Study After Left Atrial Appendage Occlusion), and ANDES trial (Short-Term Anticoagulation Versus Antiplatelet Therapy for Preventing Device Thrombosis Following Left Atrial Appendage Closure), including 704 patients, 368 in the DOAC group and 336 in the DAPT group (Figure, left).^2–4^ The primary outcome was device-related thrombosis (DRT). Secondary outcomes were all-cause death, stroke, major bleeding, any bleeding, and a composite of net cardiac and cerebrovascular adverse events (NACCE), as defined in the original trials (Figure, left). We pooled the data for all these prespecified outcomes using 2-stage random-effects models and summarized the results as risk ratios (RRs) with 95% CIs. We applied the Hartung-Knapp adjustment with the Jackson modification for small standard errors as a post hoc conservative sensitivity analysis.^5^ This meta-analysis used aggregate data from published trials; therefore, institutional review board approval and informed consent were not required. Extracted data are available from the corresponding author.

**Figure.**
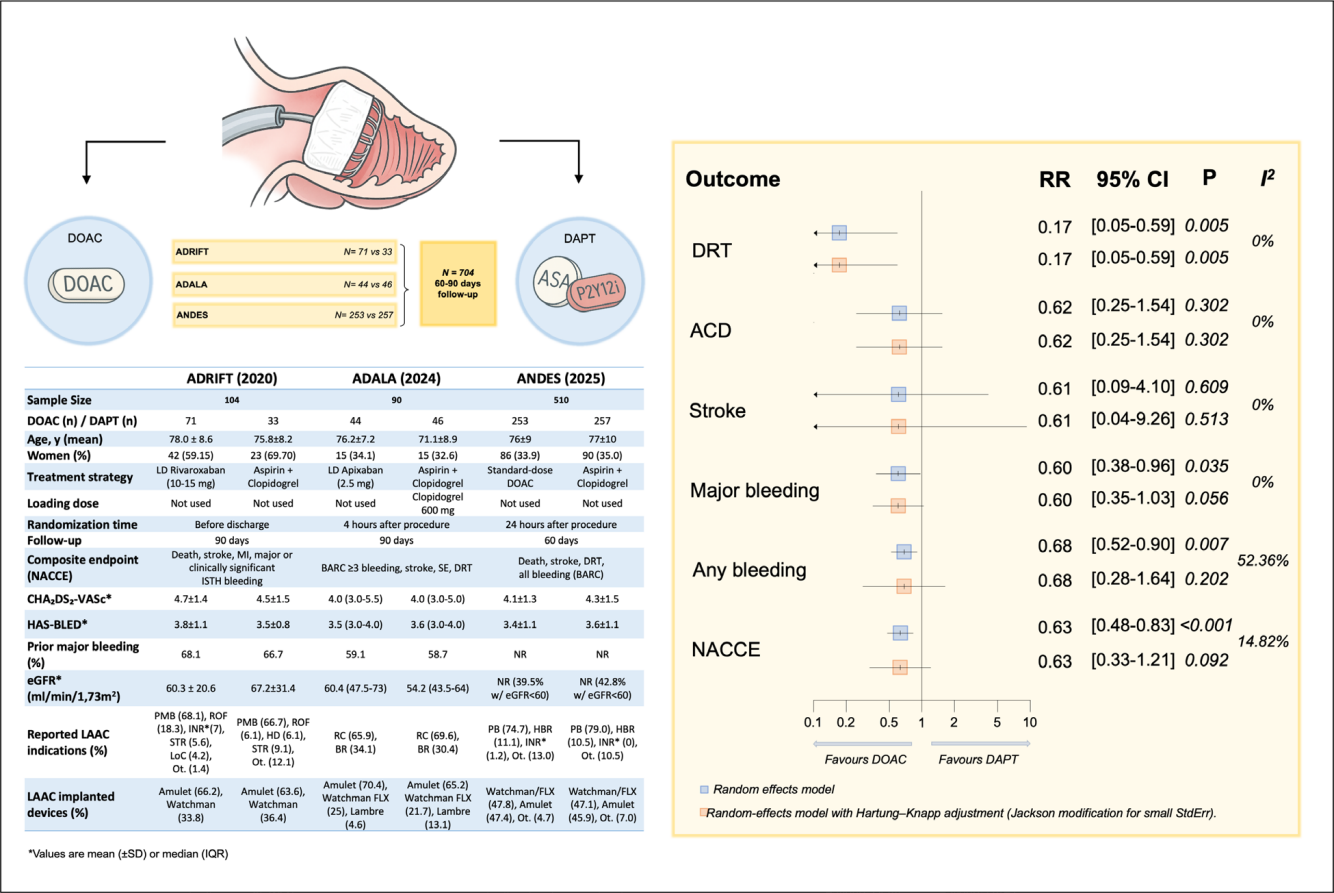
**Short-term outcomes with direct oral anticoagulant (DOAC) vs dual antiplatelet therapy (DAPT) after left atrial appendage closure (LAAC).** The **left** illustrates the contribution of each randomized trial, the pooled sample size with 60 to 90-day follow-up, key trial-level design features, and baseline clinical and procedural characteristics of the included patients. The **right** illustrates pooled RRs with 95% CIs, *P* values, and I^2^ estimates between DOAC monotherapy and DAPT for the prespecified outcomes. ACD indicates all-cause death; ADALA, Apixaban Versus Dual Antiplatelet Therapy Study After Left Atrial Appendage Occlusion; ADRIFT, Assessment of Dual Antiplatelet Therapy Versus Rivaroxaban in Atrial Fibrillation Patients Treated With Left Atrial Appendage Closure; ANDES, Short-Term Anticoagulation Versus Antiplatelet Therapy for Preventing Device Thrombosis Following Left Atrial Appendage Closure; ASA, acetylsalicylic acid; BARC, Bleeding Academic Research Consortium; BR, bleeding risk; DRT, device-related thrombosis; eGFR, estimated glomerular filtration rate; HBR, high bleeding risk; HD, hemostasis disorder; Het., heterogeneity; I^2^, inconsistency index; INR, labile international normalized ratio; IQR, interquartile range; ISTH, International Society on Thrombosis and Hemostasis; LAAC, left atrial appendage closure; LD, low-dose; LoC, lack of compliance; MI, myocardial infarction; NACCE, net adverse cardiac and cerebrovascular events; NR, not reported; Ot., other; P2Y12i, P2Y_12_ inhibitor; PB, prior bleeding; PMB, prior major bleeding; RC, relative contraindication; ROF, high risk of fall; RR, risk ratio; SD, standard deviation; SE, systemic embolism; StdErr, standard error; STR, stroke recurrence; and TEE, transesophageal echocardiography.

Main trial-level design features and baseline characteristics are summarized in Figure, left. The mean age was 76.5 years, 38.5% of patients were women, and all patients had nonvalvular atrial fibrillation. Ischemic and bleeding risks were both high, with mean congestive heart failure, hypertension, age ≥75 years (2 points), diabetes, stroke or TIA or thromboembolism (2 points), vascular disease, age 65 to 74 years, and sex category (CHA_2_DS_2_-VASc) and hypertension, abnormal renal/liver function, stroke, bleeding, labile international normalized ratio, elderly, drugs or alcohol (HAS-BLED) scores of 4.2 and 3.2, prior major bleeding in approximately 3-quarters of patients, and relative contraindications to long-term oral anticoagulation (Figure, left). After 60 to 90 days of postprocedural antithrombotic treatment, DOAC was associated with a substantial reduction in the risk of DRT compared with DAPT (RR, 0.17 [95% CI, 0.05–0.59]). DOAC was also associated with lower risks of major bleeding (RR, 0.60 [95% CI, 0.38–0.96]), any bleeding (RR, 0.68 [95% CI, 0.52–0.90]), and NACCE (RR, 0.63 [95% CI, 0.48–0.83]; Figure, right). All-cause death and stroke were not significantly different between groups. In a conservative sensitivity analysis with adjusted 95% CIs, the reduction in DRT remained statistically significant, whereas the results for major bleeding, any bleeding and NACCE were no longer significant, reflecting the limited number of trials. Between-trial heterogeneity was not significant, except for any bleeding (*I*^2^=52.36%) (Figure, right).

In aggregate, these findings suggest that in the early LAAC postprocedural period, DOAC monotherapy appears to be associated with reduced DRT compared with DAPT and possibly with lower risks of major bleeding, any bleeding, and NACCE. These clinically relevant findings, observed within a short time period, may also be less influenced by competing risks and new conditions that typically accumulate over time in patients who have undergone LAAC. Nevertheless, our results should be interpreted in light of the limited sample size and number of events. In addition, there was mild variability in the definitions of NACCE and bleeding across trials (Figure, left). Finally, different DOACs and doses were used, precluding conclusions on the optimal drug and regimen (Figure, left).

In conclusion, this meta-analysis of randomized trials indicates that short-term DOAC monotherapy after LAAC reduces DRT compared with DAPT. Major bleeding, any bleeding, and NACCE may be consistently lower with DOAC monotherapy compared with DAPT. Pending confirmation in larger, high-quality trials, these findings support the use of DOAC monotherapy over DAPT in the early post-LAAC period.

## ARTICLE INFORMATION

### Disclosures

Dr Capodanno declares speaker’s fees or honoraria from Daiichi Sankyo, Sanofi, and Terumo. The other authors report no conflicts.
